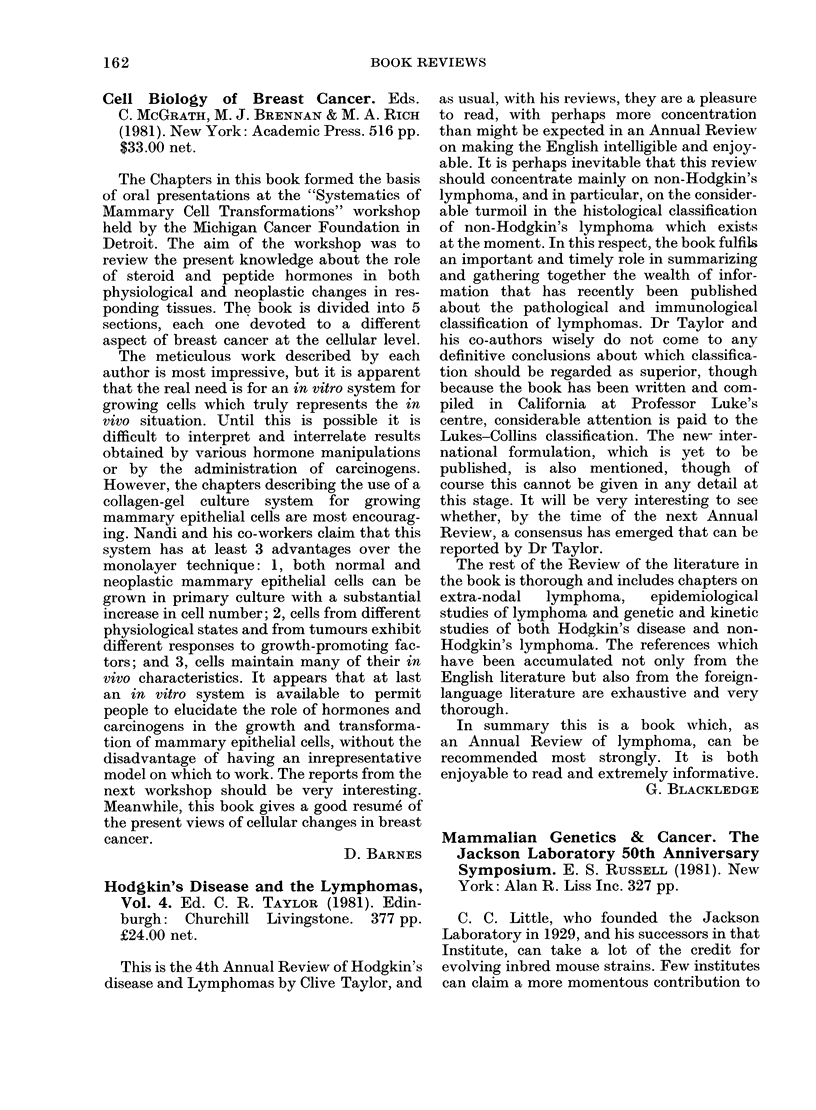# Hodgkin's Disease and the Lymphomas, Vol. 4

**Published:** 1982-01

**Authors:** G. Blackledge


**Hodgkin's Disease and the Lymphomas, Vol. 4.** Ed. C. R. Taylor (1981). Edinburgh: Churchill Livingstone. 377 pp. £24.00 net.

This is the 4th Annual Review of Hodgkin's disease and Lymphomas by Clive Taylor, and as usual, with his reviews, they are a pleasure to read, with perhaps more concentration than might be expected in an Annual Review on making the English intelligible and enjoyable. It is perhaps inevitable that this review should concentrate mainly on non-Hodgkin's lymphoma, and in particular, on the considerable turmoil in the histological classification of non-Hodgkin's lymphoma which exists at the moment. In this respect, the book fulfils an important and timely role in summarizing and gathering together the wealth of information that has recently been published about the pathological and immunological classification of lymphomas. Dr Taylor and his co-authors wisely do not come to any definitive conclusions about which classification should be regarded as superior, though because the book has been written and compiled in California at Professor Luke's centre, considerable attention is paid to the Lukes–Collins classification. The new international formulation, which is yet to be published, is also mentioned, though of course this cannot be given in any detail at this stage. It will be very interesting to see whether, by the time of the next Annual Review, a consensus has emerged that can be reported by Dr Taylor.

The rest of the Review of the literature in the book is thorough and includes chapters on extra-nodal lymphoma, epidemiological studies of lymphoma and genetic and kinetic studies of both Hodgkin's disease and non-Hodgkin's lymphoma. The references which have been accumulated not only from the English literature but also from the foreign-language literature are exhaustive and very thorough.

In summary this is a book which, as an Annual Review of lymphoma, can be recommended most strongly. It is both enjoyable to read and extremely informative.

G. Blackledge